# Supplementary material for: Prognostic Accuracy of the qSOFA Score for In-Hospital Mortality in Elderly Patients with Obstructive Acute Pyelonephritis: A Multi-Institutional Study
Source: Diagnostics (Basel). 2021 Dec 5;11(12):2277. doi: 10.3390/diagnostics11122277 (PMC8700712; doi:10.3390/diagnostics11122277)
Supplement: Supplementary file 1 [file diagnostics-11-02277-s001.zip › Figure S1 and S2.pdf]

Figure S1

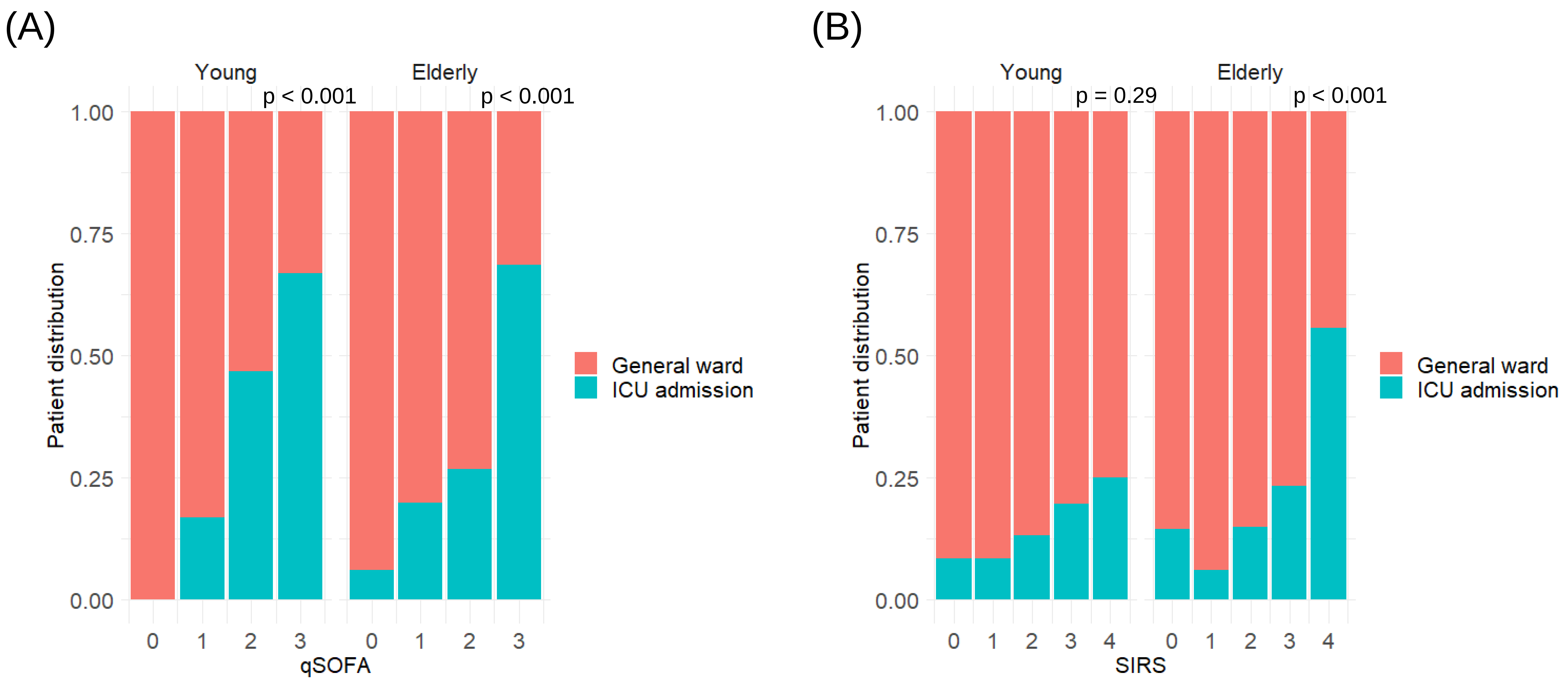

Distribution of patients who were admitted to the ICU by qSOFA and SIRS scores. A, distribution by qSOFA score in young (*left*) and elderly (*right*) patients. B, distribution by SIRS score in young (*left*) and elderly (*right*) patients.

Figure S2

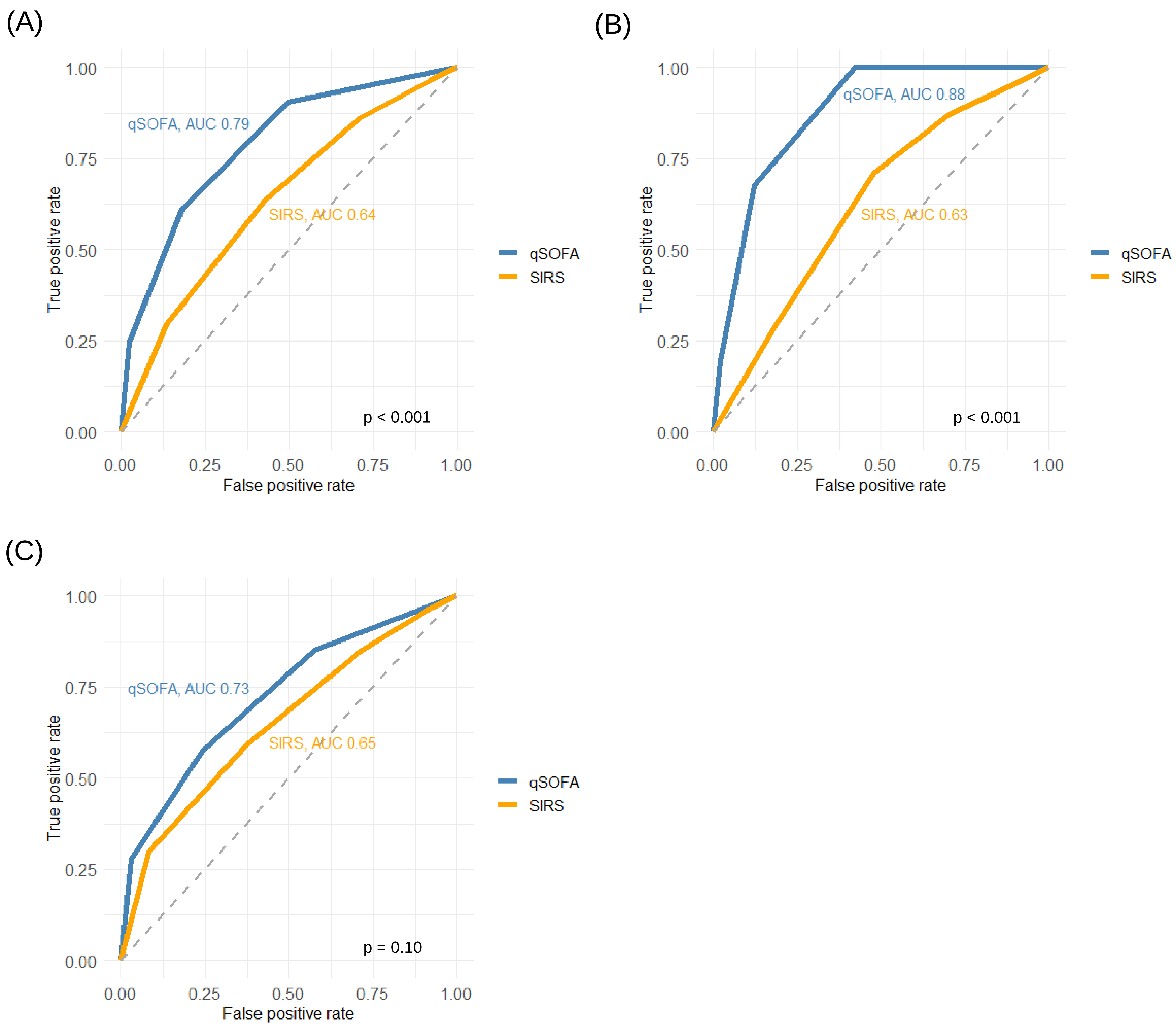

ROC curves of the qSOFA and SIRS scores in predicting the composition of ICU admission and/or in-hospital mortality in total (A), young (B), and elderly (C) patients.
