# Supplementary material for: Prognostic Accuracy of the qSOFA Score for In-Hospital Mortality in Elderly Patients with Obstructive Acute Pyelonephritis: A Multi-Institutional Study
Source: Diagnostics (Basel). 2021 Dec 5;11(12):2277. doi: 10.3390/diagnostics11122277 (PMC8700712; doi:10.3390/diagnostics11122277)
Supplement: Supplementary file 1 [file diagnostics-11-02277-s001.zip › Table S1, S2, S3 and S4.pdf]

Table S1. Comparison of baseline characteristics between included and excluded patients

Variables		Included, n (%)	Excluded, n (%)	P value
No. of patients		369 (59)	252 (41)	
Sex	Females	258 (70)	158 (63)	0.060
	Males	111 (30)	94 (37)	
Age (years), median (range)		75 (19-100)	71 (17-96)	0.002
CCI	0-1	145 (39)	159 (63)	<0.001
	≥ 2	224 (61)	93 (37)	
DM	Yes	76 (21)	54 (20)	0.80
	No	293 (79)	198 (75)	
CVD	Yes	106 (29)	46 (17)	0.003
	No	263 (71)	206 (78)	
Stroke	Yes	65 (18)	30 (11)	0.052
	No	304 (82)	222 (84)	
Malignancy	Yes	71 (19)	40 (15)	0.28
	No	298 (81)	212 (80)	
Immunosuppression	Yes	26 (7)	12 (5)	0.24
	No	343 (93)	240 (95)	
WBC (10^3/μl), median (range)		12.4 (0.8-61.5)	11.5 (1.2-39.4)	0.15
Platelet (10^4/μl), median (range)		15.7 (0.2-57.0)	19.6 (1.6-66.4)	<0.001
CRP (mg/dl), median (range)		14.3 (0.0-52.1)	10.6 (0-41.5)	<0.001
Albumin (g/dl), median (range)		3.1 (1.5-4.9)	3.5 (1.7-4.8)	<0.001
Creatinine (mg/dl), median (range)		1.2 (0.3-8.0)	1.2 (0.3-10.9)	0.81
Midstream urine culture	Positive	279 (76)	164 (65)	0.001
	Negative	80 (22)	67 (27)	
	None	10 (3)	21 (8)	
Blood culture	Positive	143 (39)	57 (23)	<0.001
	Negative	160 (43)	59 (23)	
	None	66 (18)	136 (54)	
Position of obstruction	Renal calyx or pelvis	9 (2)	5 (2)	<0.001
	Pelvic ureteral junction	53 (14)	24 (10)	
	Upper ureter	168 (45)	103 (41)	
	Mid ureter	47 (13)	22 (9)	
	Lower ureter	82 (22)	63 (25)	
	Unknown	10 (3)	34 (14)	
Laterality	Right	165 (45)	108 (45)	0.15
	Left	194 (53)	120 (50)	
	Bilateral	10 (3)	14 (6)	
Hydronephrosis	Low grade (0-2)	219 (59)	115 (46)	0.001
	High grade (3-4)	145 (40)	129 (51)	
	Unknown	4 (1)	8 (3)	
Cause of obstruction	Calculus	344 (93)	205 (81)	<0.001
	Tumor	6 (2)	8 (3)	
	Others	15 (4)	38 (15)	
	Unknown	4 (1)	1 (0)	
Method of drainage	Nephrostomy	118 (32)	19 (8)	<0.001
	Ureteral stent	186 (50)	163 (65)	
	None	65 (18)	70 (28)	
DIC	Yes	93 (25)	31 (12)	<0.001
	No	276 (75)	221 (88)	
ICU admission	Yes	70 (19)	5 (2)	
	No	299 (81)	246 (98)	
In-hospital mortality	Yes	25 (7)	2 (1)	
	No	344 (93)	249 (99)	
SIRS, median (range)		2 (0-4)	1 (0-4)	<0.001

Values are presented as number (%) or median (range). CCI, Charlson comorbidity index; CRP, C-reactive protein; CVD, cerebrovascular disease; DIC, Disseminated Intravascular Coagulation; DM, diabetes mellitus; ICU, intensive care unit; SIRS, systemic inflammatory response syndrome; WBC, White Blood Cell.

Table S2. Logistic regression analysis for the composite of ICU admission and/or in-hospital mortality

Variables		Univariate	Multivariate		
		P value	OR	95%CI	P value
Sex	Males vs. females	0.49			
Age	Elderly vs young patients	0.013			
CCI	≥ 2 vs. 0-1	0.001			
DM	Yes vs. No	0.65			
CVD	Yes vs. No	0.077			
Stroke	Yes vs. No	0.20			
Malignancy	Yes vs. No	0.67			
Immunosuppression	Yes vs. No	1.00			
Cause of obstruction	Calculus vs. Others	0.79			
	Tumor vs. Others	0.86			
Laterality	Right vs. Left	0.46			
Hydronephrosis	Low grade (0-2) vs High grade (3-5)	0.22			
CRP	≥ 14.3 vs. < 14.3	0.038			
Platelet	< 15.7 vs. ≥ 15.7	< 0.001	2.8	1.6-5.0	< 0.001
WBC	≥ 12.4 vs. < 12.4	0.78			
Cre	≥ 1.2 vs. < 1.2	< 0.001	1.9	1.1-3.4	0.024
Drainage	Nephrostomy vs. None	0.027			
	Ureteral stent vs. None	0.005			
SIRS	≥ 1 vs. 0	0.12			
qSOFA	≥ 2 vs. 0-1	< 0.001	5.7	3.3-10.0	< 0.001

CCI, Charlson comorbidity index; CI, confidence interval; CRP, C-reactive protein; CVD, cerebrovascular disease; DM, diabetes mellitus; ICU, intensive care unit; OR, odds ratio; qSOFA, quick Sequential Organ Failure Assessment; SIRS, systemic inflammatory response syndrome; WBC, white blood cell

Table S3. Logistic regression analysis for in-hospital mortality in elderly patients

Variables		Univariate	Multivariate		
		P value	OR	95%CI	P value
Sex	Males vs. females	0.76			
CCI	≥ 2 vs. 0-1	0.006	8.1	1.6-147.4	0.008
DM	Yes vs. No	0.47			
CVD	Yes vs. No	0.96			
Stroke	Yes vs. No	0.52			
Malignancy	Yes vs. No	0.41			
Immunosuppression	Yes vs. No	0.18			
Cause of obstruction	Calculus vs. Others	0.93			
	Tumor vs. Others	0.44			
Laterality	Right vs. Left	0.99			
Hydronephrosis	Low grade (0-2) vs High grade (3-5)	0.70			
CRP	≥ 14.3 vs. < 14.3	0.73			
Platelet	< 15.7 vs. ≥ 15.7	0.31			
WBC	≥ 12.4 vs. < 12.4	0.52			
Cre	≥ 1.2 vs. < 1.2	0.37			
Drainage	Nephrostomy vs. None	0.58			
	Ureteral stent vs. None	0.63			
SIRS	≥ 1 vs. 0	0.070			
qSOFA	≥ 2 vs. 0-1	0.11			
qSOFA	≥ 3 vs. 0-2	0.037	3.6	1.0-11.3	0.047

CCI, Charlson comorbidity index; CI, confidence interval; CRP, C-reactive protein; CVD, cerebrovascular disease; DM, diabetes mellitus; OR, odds ratio; qSOFA, quick Sequential Organ Failure Assessment; SIRS, systemic inflammatory response syndrome; WBC, white blood cell

Table S4. List of 25 deceased patients with OAPN

No.	Age	Sex	CCI	Drainage	qSOFA	SIRS	Cause of mortality
1	85	Male	3	None	3	4	OAPN
2	83	Femaleee	5	Ureteral stent	3	4	OAPN
3	64	Female	2	Nephrostomy	3	4	OAPN
4	81	Female	4	Nephrostomy	3	3	OAPN
5	88	Male	3	Ureteral stent	3	3	Pneumonia
6	83	Female	6	Nephrostomy	3	2	OAPN
7	88	Male	2	Ureteral stent	2	4	Pneumonia
8	74	Female	4	None	2	3	OAPN
9	87	Male	2	Nephrostomy	2	2	Pneumonia
10	75	Female	5	Ureteral stent	2	2	OAPN
11	94	Female	3	None	2	2	OAPN
12	73	Female	1	Ureteral stent	2	2	OAPN
13	91	Female	3	None	2	2	OAPN
14	71	Female	4	Nephrostomy	2	1	OAPN
15	43	Male	3	Ureteral stent	1	3	OAPN
16	80	Female	6	None	1	3	Liver failure
17	75	Male	5	Ureteral stent	1	3	Malignancy
18	78	Female	5	Ureteral stent	1	2	OAPN
19	84	Female	6	Ureteral stent	1	2	Cardiovascular
20	97	Female	2	Ureteral stent	1	1	Pneumonia
21	84	Female	3	Ureteral stent	0	3	OAPN
22	82	Female	0	Ureteral stent	0	2	OAPN
23	82	Male	8	Ureteral stent	0	1	OAPN
24	92	Female	6	Ureteral stent	0	1	Pneumonia
25	91	Female	5	Nephrostomy	0	1	Pneumonia

CCI, Charlson comorbidity index; OAPN, obstructive acute pyelonephritis; qSOFA, quick Sequential Organ Failure Assessment; SIRS, systemic inflammatory response syndrome
